# Variants of the long control region and the E6 oncogene in European human papillomavirus type 16 isolates: implications for cervical disease

**DOI:** 10.1038/sj.bjc.6600024

**Published:** 2002-01-21

**Authors:** C Kämmer, M Tommasino, S Syrjänen, H Delius, U Hebling, U Warthorst, H Pfister, I Zehbe

**Affiliations:** Institute of Virology, University of Cologne, Fürst-Pückler-Strasse 56, D-50935 Cologne, Germany; Angewandte Tumorvirologie, Deutsches Krebsforschungszentrum, Im Neuenheimer Feld 242, D-69120 Heidelberg, Germany; Department of Oral Odontology and Dentistry, University of Turku, FIN-20520 Turku, Finland

**Keywords:** human papillomavirus 16, viral variant, long control region, E6 oncogene, cervical disease

## Abstract

High-risk human papillomavirus types, especially type 16, are risk factors for cervical cancer. Preliminary studies suggest that HPV16 polymorphisms in the long control region or in the E6 gene may alter the oncogenic potential of the virus. This could partially explain why some lesions progress to cancer while others do not. A systematic study combining the long control region and E6 has not been undertaken. This prompted us to investigate the long control region and the E6 in northern European women infected with human papillomavirus 16. We identified the sequence variations of both regions and investigated the long control region promoter activity among various isolates. In addition, we correlated the distribution of long control region and E6 polymorphisms with disease status. We analyzed 45 samples from Swedish and Finnish women. The long control region and the E6 gene were sequenced after polymerase chain reaction long control region fragments of six European isolates covering the majority of polymorphisms in this region were ligated into the pALuc vector and used for luciferase assays. In European HPV16 isolates, polymorphisms in the long control region are more frequent than in the E6 gene. Nevertheless, the promoter function was slightly increased in only one of the tested European long control region variants. In addition, we found a specific European E6 variant, L83V, to be enriched in high-grade lesions and cancer rather than a specific European long control region variant. The difference in oncogenicity between European HPV16 genotypes is more probably due to an altered property of the corresponding E6 proteins rather than to an altered activity of the P97 promoter.

*British Journal of Cancer* (2002) **86**, 269–273. DOI: 10.1038/sj/bjc/6600024 www.bjcancer.com

© 2002 The Cancer Research Campaign

## 

High-risk human papillomaviruses (HPVs), especially of type 16, are considered to be the most important risk factors in the development of cervical neoplasias (zur Hausen, 1991; Walboomers *et al*, 1999). However, only a fraction of women with HPV16-positive cervical precursor lesions develop cancer. The viral oncoproteins E6 and E7 are responsible for the malignant transformation of cervical epithelium by interacting with cell cycle-regulating proteins e.g. p53 and pRb (Scheffner *et al*, 1990; Phelps *et al*, 1992). The transcription of E6 and E7 genes is controlled by the P97 promoter located in the E6 proximal part of the long control region (LCR), which contains the keratinocyte specific enhancer as well as several binding sites for cellular and viral transcription factors (Chong *et al*, 1991). Sequence variations within the LCR and/or E6 and E7 genes may therefore have an impact on the oncogenic potential of the virus.

The HPV16 genome is polymorphic with respect to geographical regions giving rise to European, African, Asian, Asian-American and north American subtypes. These can be further divided into multiple genotypes (Yamada *et al*, 1997). The originally identified reference sequence, the so-called prototype (Seedorf *et al*, 1985), belongs to the European subtype (Yamada *et al*, 1997). It was reported that the promoter activity of Asian-American and north American HPV16 variants is increased three-fold as compared to the prototype (Kämmer *et al*, 2000) and that polymorphisms in the E6 gene but not in the E7 gene correlate with the severity of the lesion in several European populations (Zehbe *et al*, 1998a,b). So far, no investigation has systematically compared LCR sequence variations with the transcriptional efficiency of the LCR promoter in combination with E6 polymorphisms and their association with cervical disease status in a larger group of clinical samples.

The objective of this study was to determine the LCR and the E6 sequences of HPV16 from north European women and to establish whether there are differences in promoter activity between the genotypes thus identified. In addition the distribution of polymorphisms in the LCR and in the E6 was correlated with disease status. We found polymorphisms in the LCR to be more common than polymorphisms in the E6 gene in European isolates. However, the promoter function was similar in six representative isolate types, except for one. In addition, no specific European LCR variant was associated with progression or higher lesion grade, while an E6 variant was found to be enriched in high-grade lesions and cancer. This E6 variant showed an amino acid change from leucine to valine at residue 83 (L83V). We conclude that the different oncogenicity between European HPV16 genotypes is more likely to be caused by an altered property in the respective E6 protein rather than by an altered activity of the P97 promoter.

## SUBJECTS AND METHODS

### Subjects

#### Clinical samples

Altogether 45 HPV16-positive specimens from 20 Swedish and 25 Finnish women were included in the study. The Swedish samples are derived from a cross-sectional study consisting of extracted DNA from formalin-fixed cervical biopsies diagnosed as having low-grade or high-grade cervical intraepithelial neoplasia (LCIN, HCIN) or invasive cervical carcinoma (ICC) (Zehbe *et al*, 1998b, 2001a). The Finnish samples are derived from a follow-up study consisting of DNA from frozen cervical biopsies with known endpoint diagnosis, i.e. LCIN or HCIN (Kurvinen *et al*, 2000). Consequently, the analysis of the present investigation of the E6 gene was performed on all samples where sufficient material was still available. Of the Swedish women, the E6 sequences have previously been reported (Zehbe *et al*, 1998b, 2001a) and of the Finnish women the LCR sequences have previously been described (Kurvinen *et al*, 2000).

### Methods

#### PCR, sequencing, and plasmid constructs of the LCRs

The LCR region from the Swedish samples was amplified by nested PCR (LCR-6933 5′-CACCTCCAGCACCTAAAGAAGATCCCC-3′, LCR-140 5′-GCAGCTCTGTGCATAACTGTGG-3′, LCRHind-7010 5′-ATCAAGCTT GACC TAGAT CAGTTT CCTTTA GGAC-3′, LCR Bam-124 5′-ATCGGATCCTCCTGTGGGTCCTGAAACATTGCAG-3′) generating fragments ranging from nucleotide (nt) positions 7010 to 124 with the inner primers containing *Hind*III and *Bam*HI restriction sites (Dong *et al*, 1994). The purified LCR fragments were ligated into the precut vector pALuc (Dong *et al*, 1994), so that the P97 promoter of the LCRs was placed in front of the luciferase reporter gene. Before using them in luciferase assays the different pALuc-clones were sequenced.

#### PCR and sequencing of the HPV 16 E6

The HPV16 E6-specific PCR amplifying the region spanning nucleotides (nts) 52–575 and the sequencing reactions of the Finnish samples were performed as described earlier (Zehbe *et al*, 1998b).

#### Cell culture and functional assays of the LCR constructs

The HPV-negative human cervical carcinoma cell line C33A (ATCC HTB-31) was used for luciferase experiments performed as described earlier (Kämmer *et al*, 2000).

#### Site directed mutagenesis with the LCR constructs

Site directed mutagenesis was performed with the US56 clone using the QuickChange Site-Directed Mutagenesis-Kit supplied by Stratagene. The nucleotide at position 78 (US56) was changed from the isolate sequence into the European reference sequence (m-US56). The sequence variation of the US56 isolate at position 78 was introduced into the European reference sequence (m-E/US56). The primers used comprised 34 bp from nt 57–89 and contained the intended nucleotide exchange. After obtaining positive clones the whole LCRs were sequenced to confirm the mutated nucleotide in the context of the former LCR sequences.

## RESULTS

### The LCR is more polymorphic than the E6 gene in European HPV16 genotypes

**Figure 1:**
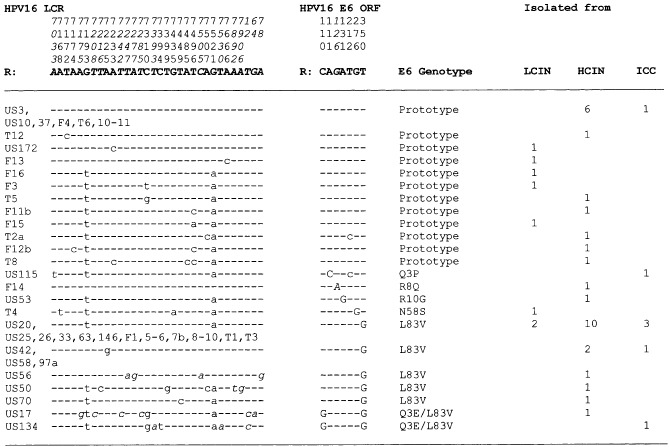
Sequence alterations relative to the LCR and E6 open reading frame of reference HPV16. Differences between the isolates and the HPV16 reference sequence (**bold**) are shown. The type of lesion from which the isolates are derived is indicated. Dashes indicate nucleotides identical to the reference sequence. Capital letters indicate alterations in the E6 open reading frame that result in amino acid changes, while lower case letters indicate silent mutations. The position of amino acid change is indicated numerically. The letter preceding this number refers to the prototype amino acid, and the letter following it refers to its substitution. The polymorphisms not reported previously appear in *italics*. Swedish cases are denoted ‘US’ and Finnish cases ‘F’ or ‘T’. HPV=human papillomavirus; LCR=long control region; ORF=open reading frame; R=reference sequence (Seedorf *et al*, 1985); L/HCIN=low-grade/high-grade cervical intraepithelial neoplasia.

After PCR, the entire HPV16 LCR (nts 7010–124) and the entire HPV16 E6 gene (nts 83–559) were sequenced and compared with the European reference sequence (HPV16R sequence in the HPV database). The sequences of all samples tested in the present investigation are summarized in Figure 1

. All 45 isolates could be assigned to the European group (Yamada *et al*, 1997). The LCR of European genotypes is more polymorphic than the E6 gene. In the LCR, 32 nt substitutions were characterized, while in the E6 gene, seven different polymorphisms were identified, six of them leading to amino acid changes. The distribution of LCR and E6 polymorphisms in HPV16-infected Finnish women resembles approximately that found in the Swedish population (Zehbe *et al*, 1998b). In the LCR the nt changes at positions 7193 and 7521 (51%) were most frequently detected and in the E6 gene, L83V was most frequently detected (67%). LCR-7193/7521 and E6-L83V were found together in 15 cases (33%) and prototype sequences in both regions were present in seven cases (16%). Consequently, 23 cases (51%) showed a linkage disequilibrium between the E6 and LCR sequences.

### Alteration in promoter activity among European LCR genotypes is a rare event

We next performed functional studies to elucidate whether specific HPV16 LCR genotypes, detected in north European women, differ in their promoter activity. Most isolates in our tested group contained polymorphisms in their LCR sequence as compared with the reference sequence (Figure 1). Some of these were identified within transcription factor-binding sites, which might modulate viral promoter activity positively or negatively (Table 1

**Table 1:**
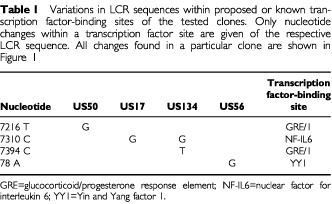
Variations in LCR sequences within proposed or known transcription factor-binding sites of the tested clones. Only nucleotide changes within a transcription factor site are given of the respective LCR sequence. All changes found in a particular clone are shown in Figure 1

) (Kurvinen *et al*, 2000). To examine a representative set of samples, we included the frequently detected LCR-7193/7521 (US53, identical with isolate 59 of Veress *et al* (1999)) as well as samples with additional changes in transcription factor-binding sites, i.e. US50, 17 and 134 (Table 1). In addition, US56 was included since it contains a variation, which has not previously been described, namely at nt 78 (Table 1). Finally, we tested US58 because the change at position 7225 was found three times in our analysis (US42, 58 and 97a; Figure 1).

**Figure 2:**
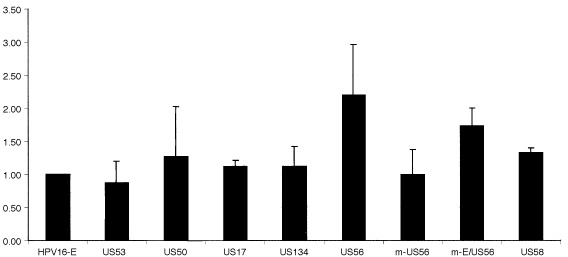
Luciferase expression under the control of the HPV16 LCR-fragments of the different European isolates The results for the isolates US53, 50, 17, 134, 56 and 58 as well as the mutated LCR m-US56 are shown. The mean values of six independent assays together with their standard deviations are demonstrated. The LCR of prototype HPV16 was used as a reference clone and set to the value 1.0 in all assays.

The HPV16 LCRs were cloned into the pALuc vector in front of the luciferase gene in order to determine whether the above-mentioned sequence alterations affect the P97 promoter activity. C33A cells were transfected with the isolate constructs and luciferase activities were analyzed 48 h later. All isolates, except US56, exhibited approximately the same activity as the European reference clone with transcription activities ranging from 0.87 to 1.27 (Figure 2

). The US56 isolate showed a stimulation of 2.2-fold compared to the European reference clone (Figure 2).

To determine whether the newly identified sequence alteration at position 78 (US56) was responsible for the 2.2-fold increase in P97 promoter activity, site directed mutagenesis was performed. US56 exhibited altogether four sequence variations but only one singular to this clone, namely the G>A transition at nt position 78. This position was mutated back into the European reference sequence, creating m-US56. The same nucleotide variation was introduced into the European reference clone creating m-E/US56. Subsequently luciferase assays were performed with the variants and their mutated counterparts. The transcriptional activity of the P97 promoter of m-US56 dropped to a level comparable with the European reference, whereas m-E/US56 exhibited an increase (1.73), thus indicating that indeed this nucleotide exchange is important for the regulation of the P97 promoter activity (Figure 2).

Together, these and previously published results clearly show that most LCR polymorphisms do not cause a change in promoter activity. Indeed, when considering all LCR isolates tested thus far (Veress *et al*, 1999) only one of a total of eight identified European LCR variants revealed an increased promoter activity compared to the prototype. Interestingly, the change in promoter activity could be singled out to just one nt exchange being an A to G change at position 78.

### Variations in the E6 gene but not in the LCR of European HPV16 genotypes correlate with disease status

We next determined, whether the most frequently detected genotypes, LCR-7193/7521 and E6-L83V, correlate with disease status. No change in promoter activity has been observed with LCR-7193/7521 compared to the prototype. With these results in hand, one would not expect LCR-7193/7521 to correlate with disease status. E6-L83V, on the other hand, has previously been shown to be associated with high-grade lesions and cancer in Swedish women and Norwegian women (Zehbe *et al*, 1998b; Andrew Jenkins, personal communication). In the Finnish group, E6-L83V was associated with progression in 50% of the cases, whereas the same genotype was present in only 20% of the regressing lesions. In contrast, the prevalence of 7193/7521 irrespective of the E6 polymorphism was similar in regressing and in progressing lesions with known endpoint diagnosis, being 60 and 67%, respectively (data not shown). Since the association of L83V with disease status is similar in Swedish and Finnish women, the data of the two groups were pooled in the current study. A statistically significant trend for an increased prevalence of E6-L83V with increasing severity of the lesion (*P*=0.04) was observed, while this was not the case for LCR-7193/7521 (*P*=0.15) (Table 2

**Table 2:**
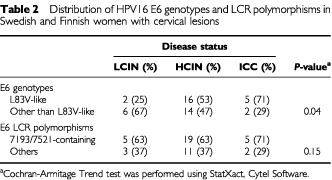
Distribution of HPV16 E6 genotypes and LCR polymorphisms in Swedish and Finnish women with cervical lesions

). These results indicate that certain E6 polymorphisms rather than LCR polymorphisms are associated with disease status in women infected with European HPV16 genotypes. This may reflect a difference in oncogenicity between European HPV16 genotypes, which is caused by an altered property of the various E6 proteins rather than to an altered activity of the P97 promoter.

## DISCUSSION

In the present study, we have established the LCR sequences of HPV16 genotypes and compared the viral P97 promoter activity in a sub-set of the isolates detected. In addition, we assessed the significance of LCR and E6 polymorphisms for the clinical outcome of cervical disease in Swedish and Finnish women. All cases of our samples could be assigned to the European subtype. The LCR region of European HPV16 genotypes is more polymorphic than the E6 gene in our samples. In half of the samples, the LCR and the E6 sequences were in linkage disequilibrium. In spite of the variability of the LCR even within known or proposed transcription factor-binding sites, LCR polymorphisms appear not to influence the fate of the disease in women infected with European variants, while we could show in the present and in previous investigations that this, indeed, is the case with specific European HPV16 E6 genotypes. This is underlined by the fact that most European LCR genotypes analyzed to date have a promoter activity comparable to the reference isolate (Veress *et al*, 1999; this study). So far, only one variation, namely at position 78 within a YY1-binding site (O'Connor *et al*, 1996) with a change from A to G was found to be responsible for a two-fold increase of the promoter activity when compared with the prototype. Interestingly, after a more extensive mutagenesis from CAT to TGC (nts 77–79), no effect on promoter activity was observed (O'Connor *et al*, 1996). This may be due to a change in binding of another factor to this site acting as a mild activator. The 78 variant was detected only once in our study group and has not previously been described in clinical samples. Notably, other alterations within transcription factor sites identified in the present investigation (Table 1) did not change promoter activity. The HPV16 E6-L83V variant, on the other hand, is the most common E6 variant in north European women and correlates statistically with the severity of cervical disease in three Scandinavian countries, i.e. Finland, Sweden and Norway (Zehbe *et al*, 1998b; Kurvinen *et al*, 2000; Andrew Jenkins, personal communication). The reason for this is probably not due to functional differences of the E6 protein but rather to the fact that this particular viral protein evades host immune recognition (Zehbe *et al*, 2001b). This is further underlined by the fact that the oncogenicity of viral variants seems to be related to the geographical area studied. For instance, E6-L83V is not enriched in Italian or Czech women with cancer. Instead, other E6-genotypes are found to be associated with cancer in these women (Zehbe *et al*, 2001b). In addition, E6-L83V is evenly distributed in precursor lesions and cancer in a German study (Nindl *et al*, 1999).

While European LCR isolates do not differ a great deal in their promoter activity, this is not the case with LCR variants of the Asian-American and north American lineages. Interestingly, all genotypes within these two branches contain the nucleotide exchange at position 7729, which is responsible for a three-fold increased activity of the P97 promoter compared to the reference isolate (Kämmer *et al*, 2000). This nt exchange is not present in African or European genotypes. A statistically significant association was observed between non-European LCR variants containing this polymorphism and disease status in a study of women from Costa Rica (Hildesheim *et al*, 2001). Similar results were obtained in a Brazilian and in a north American study (Xi *et al*, 1997; Villa *et al*, 2000). Moreover, we could show that in Italian women, the Asian-American variant had a nine-fold higher prevalence in cervical cancer than in low-grade precursor lesions (Zehbe *et al*, 2001b ) and in the Finnish group the one case infected with this variant progressed (Kurvinen *et al*, 2000).

In summary, the oncogenic potential of Asian-American or north American lineages is influenced by polymorphisms in the LCR and possibly in other viral genome regions. In contrast, in the European lineage this phenomenon appears to be associated with E6 rather than LCR variations.
